# Hydroxocobalamin-Induced Oxalate Nephropathy in a Patient With Smoke Inhalation

**DOI:** 10.1016/j.ekir.2021.05.019

**Published:** 2021-06-08

**Authors:** Jordan Evans, Abhishek Pandya, Yanli Ding, Wajeh Y. Qunibi

**Affiliations:** 1 Division of Nephrology, Department of Medicine, University of Texas Health Science Center, San Antonio, Texas

Smoke inhalation is the leading cause of death from fire-related events because it can result in thermal injury to the lungs and upper airways; chemical injury from byproducts of burned items; and poisonings, such as carbon monoxide and cyanide. Cyanide is a potent toxin that inhibits numerous enzymes, including cytochrome oxidases leading to cellular hypoxia, cardiovascular collapse, and death. Hydroxocobalamin, a natural form of vitamin B_12_ that chelates cyanide leading to formation of cyanocobalamin that is excreted by the kidneys, has been increasingly used as an antidote of cyanide poisoning. However, a retrospective French study found that hydroxocobalamin use for smoke inhalation was associated with an increased risk of acute kidney injury (AKI) and a need for renal replacement therapy.^1^ Moreover, another study reported high urine oxalate levels in patients treated with hydroxocobalamin.^2^ There is a paucity of reports regarding hydroxocobalamin-induced AKI from oxalate nephropathy. We present a case of biopsy-proven hydroxocobalamin-induced oxalate nephropathy in a patient admitted for smoke inhalation.

## Case Presentation

We present the case of a 54-year-old man with a history of alcohol abuse who was brought to the hospital after prolonged smoke exposure from a housefire. En route, he received hydroxocobalamin intravenously for suspected cyanide poisoning. On arrival, he was disoriented but hemodynamically stable and without skin burns. His initial laboratory values are listed in Table 1. His oxygen saturation was initially 90% but increased to 100% on oxygen mask. His urine was red, but urinalysis showed no proteinuria, and urine microscopy did not detect erythrocytes, leukocytes, or casts. Chest X-ray showed no abnormality. Cyanide level was undetectable, but carboxyhemoglobin level was elevated to 10.8% (normal range 0.0%–1.4%), consistent with carbon monoxide poisoning. He had carbonaceous soot around his oral mucosa and needed increased oxygen supplementation. He was intubated for airway protection due to concern for inhalational lung injury then transferred to the intensive care unit. Bronchoscopy revealed black carbonaceous material in the airways but no evidence of burn injury. In the intensive care unit, he became hypotensive and required aggressive volume resuscitation and vasopressor support. He also had metabolic acidosis with arterial pH of 7.21 and thus was started on sodium bicarbonate infusion.

**Table 1:** Laboratory data on day 1 of admission and day 3 when renal replacement therapy was initiated

Laboratory Test	Day 1 result	Day 3 result	Reference range
Sodium (mEq/l)	133	133	135–145
Potassium (mEq/l)	3.1	4.2	3.5–5.2
Chloride (mEq/l)	102	103	94–106
Bicarbonate (mEq/l)	22	19	20–28
BUN (mg/dl)	8	28	7–25
Creatinine (mg/dl)	0.86	5.93	0.60–1.30
Anion gap	9	11	8–12
Lactic acid (mEq/l)	1.4	0.9	0.5–2.0
Hemoglobin (g/dl)	14.7	12.2	12.8–17
White blood cells (× 10^9^/l)	11.3	15.4	3.4–10.4
Platelets (× 10^9^/l)	232	157	140–377
Ethanol (mg/dl)	232	—	0
Carboxyhemoglobin (%)	10.8	—	0–1.4

On Day 2, he developed AKI with serum creatinine rising from a baseline of 0.86 to 2.1 mg/dl. Renal ultrasound revealed no abnormalities. On the third hospital day, he was extubated, but renal function continued to worsen with serum creatinine rising to 5.93 mg/dl (Table 1). His urine became dark red from hydroxocobalamin use, and urine output progressively declined until he became anuric. Although he was hemodynamically stable, he was initiated on continuous renal replacement therapy rather than hemodialysis to avoid the frequent false “blood leak” alarms triggered by the red discoloration of the body fluids.^3^^,^^4^

**Figure 1:**
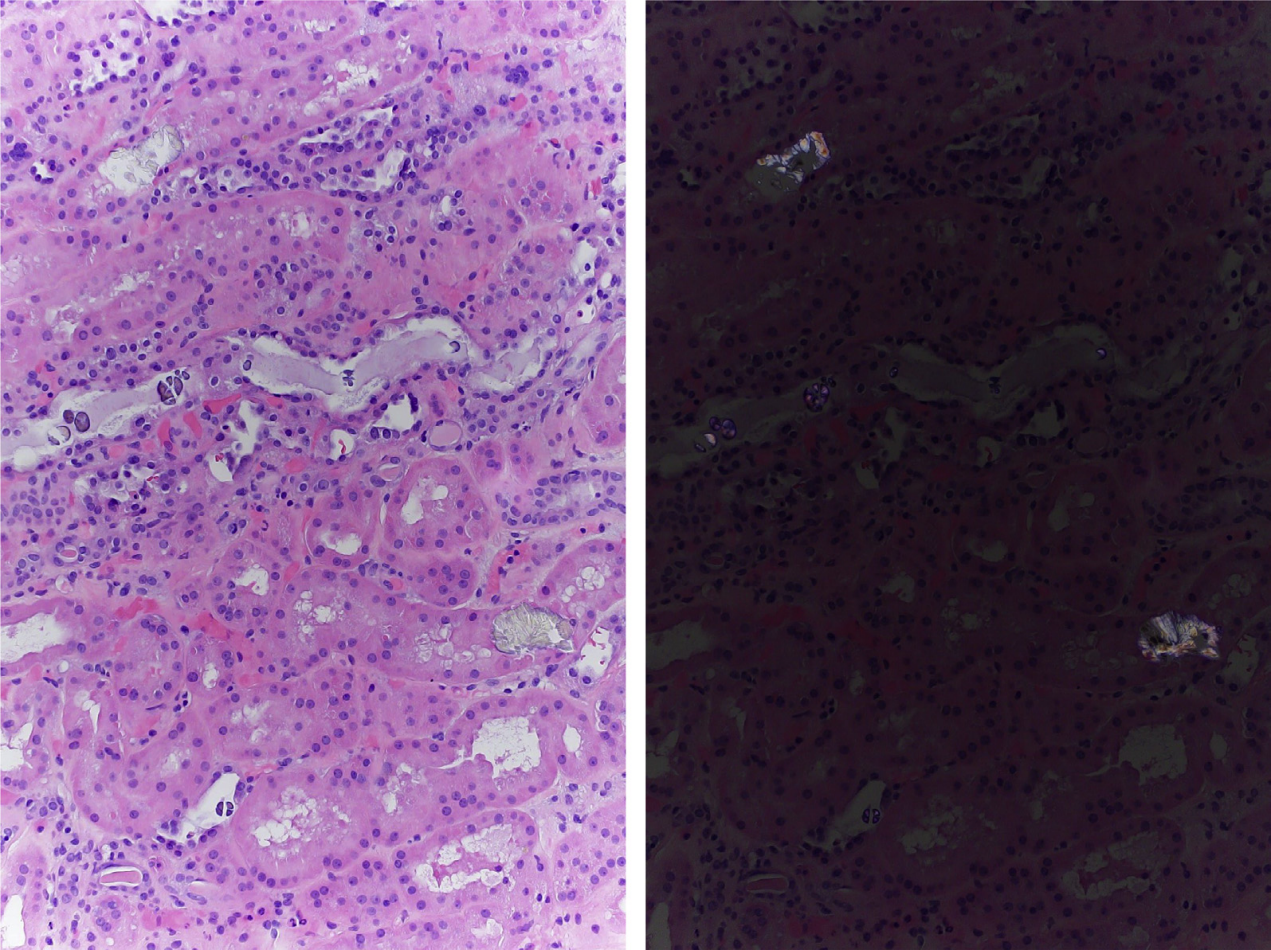
Kidney biopsy showing mild acute tubular injury and extensive tubular deposition of pale yellow calcium oxalate crystals (left, hematoxylin and eosin, original magnification ×100). These crystals are birefringent under polarized light (right, hematoxylin and eosin, original magnification ×100).

**Figure 2:**
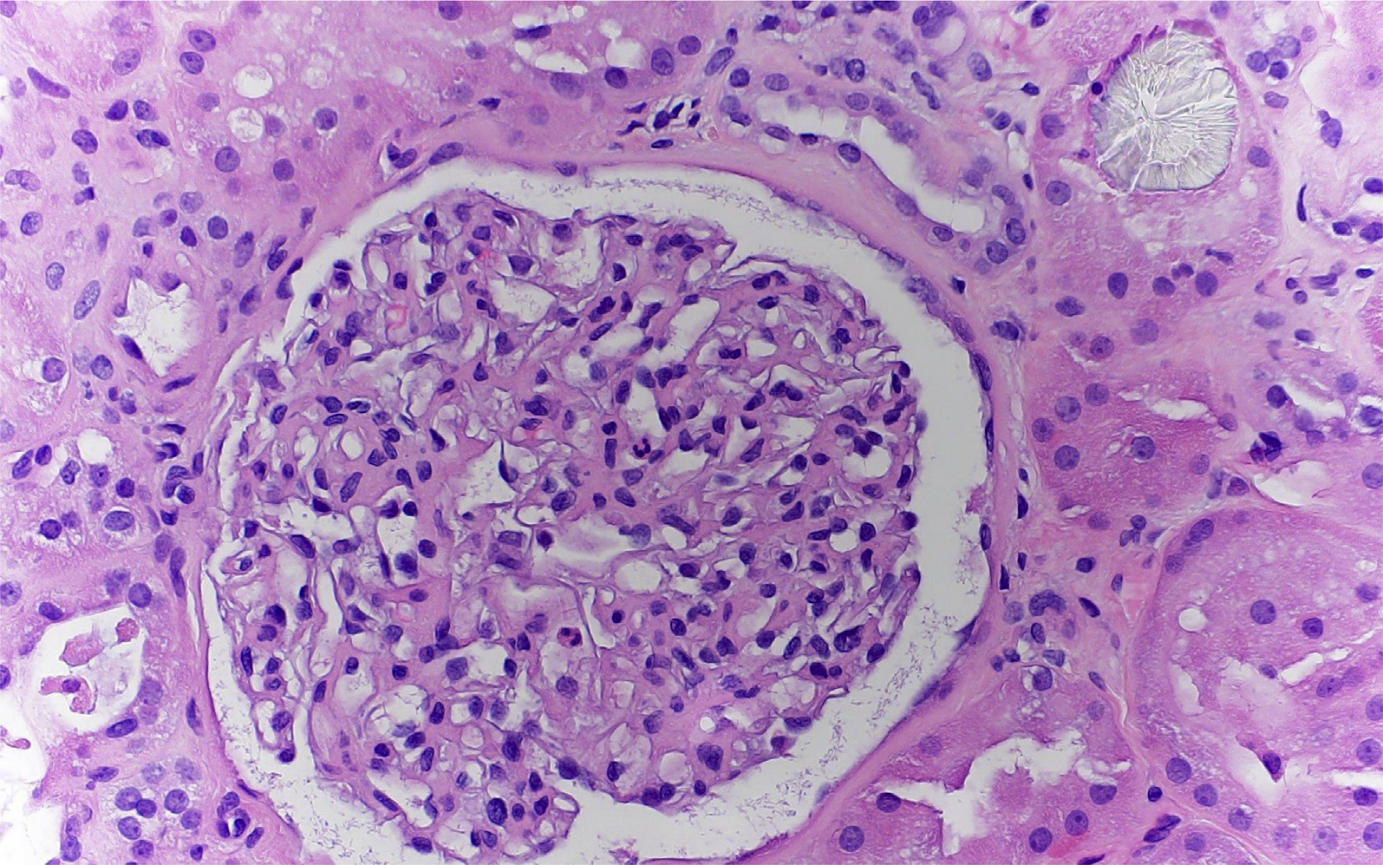
Kidney biopsy at higher magnification showing a renal tubule with calcium oxalate crystal deposition (hematoxylin and eosin, original magnification ×400).

The etiology of his AKI was not immediately clear given that he was only briefly hypotensive and was not given nephrotoxic agents. Oxalate nephropathy was suspected because his 24-hour urine oxalate was found to be 40 mg (reference range 16–49 mg/day) and serum oxalate was 22.5 mmol/L (reference range ≤ 1.9). Renal biopsy was performed on day 4 to establish the cause of his AKI. Histologic examination showed mild acute tubular necrosis and minimal interstitial fibrosis. It also showed widespread calcium oxalate crystal deposition within renal tubules with characteristic bright birefringence when viewed under polarized light (Figures 1 and 2). The patient denied any history of high oxalate diet, bariatric surgery, inflammatory bowel disease, or any medication use before his admission. Therefore, the renal biopsy findings confirmed a diagnosis of secondary oxalate nephropathy, which in our case was attributed to hydroxocobalamin administration. He was transitioned from continuous renal replacement therapy to intermittent hemodialysis on day 5. He was also prescribed a low oxalate diet, oral calcium acetate, and pyridoxine 5 mg/kg daily. His urine output improved over time, but he remained dialysis dependent at the time of his discharge. He eventually recovered from AKI, and hemodialysis was discontinued 1 month after discharge from our hospital.

## Discussion

Cyanide poisoning can occur in victims of smoke inhalation when carpeting, insulation, and plastic materials are burned. The toxicity of cyanide derives from its binding to cytochrome oxidase A3 in the electron transport chain, blocking all cellular respiration and causing massive shifts toward anerobic metabolism and lactate generation.^5^ Definitive diagnosis of cyanide poisoning can be difficult because serum levels are not readily available. Serum lactate levels can be used as a rapid surrogate marker for cyanide poisoning because serum lactate increases proportionally with the degree of cyanide poisoning.

Treatment options for cyanide poisoning historically included amyl nitrite, sodium nitrite, dicobalt edetate, and sodium thiosulfate. In December 2006, the vitamin B_12_ derivative hydroxocobalamin was approved by the US Food and Drug Administration for treatment of cyanide poisoning. Since then, hydroxocobalamin has been used as first-line therapy for patients with cyanide poisoning given its efficacy and superior safety profile. The cobalt moiety of this drug directly binds to cyanide within the cell to form cyanocobalamin, a compound easily excreted in the urine.^5^

Although generally regarded safe, widespread use of hydroxocobalamin as a first-line agent for cyanide poisoning has been questioned by some.^1^ A multicenter retrospective study from France found that hydroxocobalamin use in patients with smoke inhalation did not confer a survival advantage and was associated with increased risk of severe AKI and renal replacement therapy.^1^ The etiology of AKI in these patients was unclear because renal biopsies were not performed. In another study, hydroxocobalamin administration was associated with a significant risk of AKI and elevated urine oxalate levels in critically ill patients with burns, 2 of whom had oxalate nephropathy on renal biopsy.^2^

Our patient did not have cyanide poisoning, given that his serum cyanide level was undetectable and serum lactate was normal. However, he did receive i.v. hydroxocobalamin empirically in the field due to smoke inhalation. He had elevated serum oxalate levels and subsequently developed AKI. Renal biopsy documented oxalate nephropathy as a cause of his AKI. It is unlikely that the mild acute tubular necrosis seen in biopsy contributed to AKI. Unlike primary hyperoxaluria, which is a rare genetic disorder discovered during childhood, secondary hyperoxaluria is acquired later in life and encountered more commonly in clinical practice. Our patient fulfilled the criteria for the diagnosis of secondary oxalate nephropathy^6^ but was not exposed to causes of oxalosis other than hydroxocobalamin (Table 2). Treatment options for secondary hyperoxaluria are limited but include hydration and dietary counseling for a normal calcium, low-oxalate diet. Moreover, patients are prescribed diuretics for fluid overload, alkali therapy to alkalinize the urine, and pyridoxine to convert glyoxylate, the main precursor of oxalate, to glycine.^6^ The mechanism by which hydroxocobalamin causes oxalate nephropathy has not been fully elucidated. One plausible explanation is that hydroxocobalamin, a natural form of vitamin B_12_, acts as a cofactor in the conversion of methylmalonic acid CoA to succinyl CoA, thus providing the substrate for the formation of oxaloacetate in the citric acid cycle. Oxalate is then produced as a byproduct during the conversion of oxaloacetate to citrate.

**Table 2:** Causes of secondary hyperoxaluria

Categories	Examples
Increased oxalate precursors	Ingesting high quantities of ethylene glycol, peanuts, rhubarb, vitamin C, cashew pseudofruit
Fat malabsorption	Small bowel resection, bariatric surgery, chronic pancreatitis, systemic sclerosis, cystic fibrosis, Crohn’s disease, Celiac disease
Intestinal decolonization of oxalate-degrading bacteria species such as *Oxalobacter formigenes*	Antibiotic use, small intestine bacterial overgrowth, *Clostridium difficile* infection
Increased colonic permeability to oxalate	Inflammatory bowel disease, *C. difficile* infection
Drugs	Orlistat, octreotide, mycophenolate mofetil, hydroxocobalamin

## Conclusion

We have presented a rare case of hydroxocobalamin-induced oxalate nephropathy. Given that i.v. administration of hydroxocobalamin as a first-line treatment of cyanide poisoning is now considered standard practice in many medical centers, we suspect that cases of oxalate nephropathy such as ours may be diagnosed more frequently. Clinicians should be aware of this potentially serious complication of i.v. hydroxocobalamin and should be judicious with its empiric use in unconfirmed diagnoses of cyanide toxicity. Future studies should investigate the scope of this complication and its potential mechanisms.

## Disclosure

All the authors declared no competing interests.

## Patient Consent

The authors declare that they have obtained consent from the patient discussed in the report.
